# Supplementary material for: Analysis of PPARγ Signaling Activity in Psoriasis
Source: Int J Mol Sci. 2021 Aug 10;22(16):8603. doi: 10.3390/ijms22168603 (PMC8395241; doi:10.3390/ijms22168603)
Supplement: Supplementary file 1 [file ijms-22-08603-s001.zip › Supplemental materials_Analysis of PPARg signaling activity in psoriasis/Pathway models/Models images and html files/Interleukin-17 and interleukin-22 signaling in psoriasis/24692.html]

IL17RA


# Protein IL17RA

|  |  |
| --- | --- |
| URN | urn:agi-llid:23765 |
| Total Entities | 0 |
| Connectivity | 1046 |
| Name | IL17RA |
| Description | interleukin 17 receptor A |
| Notes | Interleukin 17A (IL17A) is a proinflammatory cytokine secreted by activated T-lymphocytes. It is a potent inducer of the maturation of CD34-positive hematopoietic precursors into neutrophils. The transmembrane protein encoded by this gene (interleukin 17A receptor; IL17RA) is a ubiquitous type I membrane glycoprotein that binds with low affinity to interleukin 17A. Interleukin 17A and its receptor play a pathogenic role in many inflammatory and autoimmune diseases such as rheumatoid arthritis. Like other cytokine receptors, this receptor likely has a multimeric structure. Alternative splicing results in multiple transcript variants encoding different isoforms. [provided by RefSeq, Feb 2014] |
| Primary Cell Localization | Plasma membrane |
| Class | Receptor |
| ObjectType | Protein |

---

|  |  |
| --- | --- |
| Pathway | Natural Killer Cell Precursor -> Natural Killer Cell Surface Expression Markers |
|  | IL17 Signaling in Psoriasis |
|  | Proteins Involved in Helicobacter Infections |
|  | Eosinophil Survival in Asthma |
|  | Il17 Signaling Related Neutrophilia in Asthma |
|  | IL17F Signaling in Bronchial Epithelial Cell in Asthma |
|  | Synovial Fibroblast Activation in Psoriatic Arthritis |
|  | Keratinocyte Activation in Psoriatic Arthritis |
|  | Proteins Involved in Psoriatic Arthritis |
|  | Proteins Involved in Endometriosis |
|  | Cytokines Trigger Otitis Media |
|  | IL23A and IL17A Provoke Cancer-Associated Inflammation |
|  | sars-cov binding |
|  | 2\_Interleukin-17 and interleukin-22 signaling in psoriasis |
|  | 2\_3\_Eosinophilia and neutrophilia in asthma: Neutrophil chemotaxis and activation |
|  | 2\_2\_Eosinophilia and neutrophilia in asthma: eosinophil apoptosis |
|  | Interleukin-17 and interleukin-22 signaling in psoriasis |

---

|  |  |
| --- | --- |
| Group | Genes with Mutations Associated with Psoriatic Arthritis |
|  | binding partnetrs for SARSs |

---

|  |  |
| --- | --- |
| MedScan ID | 23765 |

---

|  |  |
| --- | --- |
| LocusLink ID | 23765 |
|  | 16172 |
|  | 312679 |

---

|  |  |
| --- | --- |
| Alias | Cdw217 |
|  | IL17 receptors |
|  | IL17R |
|  | interleukin 17 receptor A |
|  | interleukin IL17 receptors |
|  | LOC150166 |
|  | IL17 receptor |
|  | VDw217 |
|  | interleukin IL17A receptor |
|  | HIL-17R |
|  | GNB:2826476 |
|  | interleukin IL17 receptor A |
|  | CANDF5 |
|  | interleukin IL17R |
|  | interleukin IL17 receptor |
|  | IL17RA |
|  | LOC100506204 |
|  | MGC10262 |
|  | AW538159 |
|  | interleukin IL17RA |
|  | interleukin 17 receptor |
|  | CD217 |
|  | IMD51 |
|  | IL-17 receptor A |
|  | interleukin 17A receptor |
|  | interleukin 17 receptors |
|  | IL-17RA |
|  | interleukin-17 receptor A |

---

|  |  |
| --- | --- |
| GO ID | 0030368 |
|  | 0005102 |
|  | 0007166 |
|  | 0050832 |
|  | 0072537 |
|  | 0071621 |
|  | 0097400 |
|  | 1900017 |
|  | 2000667 |
|  | 0032747 |
|  | 2000664 |
|  | 0005576 |
|  | 0005887 |
|  | 0005886 |
|  | 0071345 |
|  | 0050729 |
|  | 0016021 |
|  | 0016020 |
|  | 0019221 |

---

|  |  |
| --- | --- |
| KEGG ID | hsa:23765 |
|  | mmu:16172 |

---

|  |  |
| --- | --- |
| Organism | Homo sapiens {Organism urn:agi-taxid:9606} |
|  | Mus musculus {Organism urn:agi-taxid:10090} |
|  | Rattus norvegicus {Organism urn:agi-taxid:10116} |
|  | Homo sapiens |
|  | Mus musculus |
|  | Rattus norvegicus |

---

|  |  |
| --- | --- |
| Mouse chromosome position | 6 56.95 cM |

---

|  |  |
| --- | --- |
| OMIM ID | 605461 |
|  | 613953 |

---

|  |  |
| --- | --- |
| Rat chromosome position | 4q42 |

---

|  |  |
| --- | --- |
| Hugo ID | 5985 |
|  | HGNC:5985 |

---

|  |  |
| --- | --- |
| Human chromosome position | 22q11.1 |

---

|  |  |
| --- | --- |
| Swiss-Prot Accession | Q96F46 |
|  | Q96F46.2 |
|  | Q60943 |
|  | Q60943.1 |
|  | D4A740 |
|  | O43844 |
|  | Q20WK1 |

---

|  |  |
| --- | --- |
| GenBank ID | NC\_000022 |
|  | NM\_014339 |
|  | NP\_055154 |
|  | NM\_001289905 |
|  | NP\_001276834 |
|  | NG\_028257 |
|  | AC006946 |
|  | CH471193 |
|  | EAW57738 |
|  | EAW57739 |
|  | EAW57740 |
|  | EAW57741 |
|  | CS399134 |
|  | CAL44890 |
|  | GM832013 |
|  | CAV28582 |
|  | JA381715 |
|  | CCB07364 |
|  | AF021799 |
|  | AK056836 |
|  | AK093772 |
|  | BC011624 |
|  | AAH11624 |
|  | BC012071 |
|  | BE242733 |
|  | BU585070 |
|  | BX334547 |
|  | BX368715 |
|  | BX410574 |
|  | CB044543 |
|  | CT841520 |
|  | CAJ86450 |
|  | JF305973 |
|  | ADY18334 |
|  | U58917 |
|  | AAB99730 |
|  | Q96F46 |
|  | NC\_000072 |
|  | XM\_006505618 |
|  | XP\_006505681 |
|  | NM\_008359 |
|  | NP\_032385 |
|  | NT\_039382 |
|  | AC007844 |
|  | AC018559 |
|  | AC078896 |
|  | CH466523 |
|  | EDK99637 |
|  | EDK99638 |
|  | AK010040 |
|  | BAB26660 |
|  | AK036587 |
|  | AK080226 |
|  | BC060219 |
|  | BY267308 |
|  | U31993 |
|  | AAC52357 |
|  | Q60943 |
|  | NC\_005103 |
|  | NM\_001107883 |
|  | NP\_001101353 |
|  | AC\_000072 |
|  | AABR07061911 |
|  | AAHX01032648 |
|  | CH473964 |
|  | EDM02034 |
|  | EDM02035 |
|  | FQ225026 |
|  | FQ235042 |
|  | NC\_018933 |
|  | AMYH02037985 |
|  | AMYH02037986 |
|  | DQ891962 |
|  | ABM82888 |
|  | DQ895152 |
|  | ABM86078 |
|  | XM\_006507122 |
|  | XP\_006507185 |
|  | AC\_000028 |
|  | AAHY01057029 |
|  | BC146306 |
|  | AAI46307 |
|  | BC156651 |
|  | AAI56652 |
|  | AC\_000154 |
|  | ABBA01001459 |

---

|  |  |
| --- | --- |
| Swiss-Prot ID | I17RA\_HUMAN |
|  | I17RA\_MOUSE |

---

|  |  |
| --- | --- |
| Cell Localization | Cell membrane |
|  | Secreted |
|  | Membrane |
|  | Plasma membrane |

---

|  |  |
| --- | --- |
| Ensembl ID | ENSG00000177663 |
|  | ENSP00000479970.1 |
|  | ENST00000612619.1 |
|  | ENSP00000320936.6 |
|  | ENST00000319363.10 |
|  | ENSMUSG00000002897 |
|  | ENSMUSP00000002976.3 |
|  | ENSMUST00000002976.4 |
|  | ENSRNOG00000011153 |
|  | ENSRNOP00000014885.6 |
|  | ENSRNOT00000014885.6 |
|  | ENSP00000479970 |
|  | ENST00000612619 |
|  | ENSP00000320936 |
|  | ENST00000319363 |
|  | ENSMUSP00000002976 |
|  | ENSMUST00000002976 |
|  | ENSRNOP00000014885 |
|  | ENSRNOT00000014885 |

---

|  |  |
| --- | --- |
| MGI ID | MGI:107399 |

---

|  |  |
| --- | --- |
| RGD ID | 1306559 |

---

|  |  |
| --- | --- |
| Unigene ID | Hs.48353 |
|  | Mm.4481 |
|  | Rn.106209 |

---

|  |  |
| --- | --- |
| Homologene ID | 7378 |

---

|  |  |
| --- | --- |
| Shape | Stick-vertex |

---
